# Difference between Outcome of Left Circumflex Artery and Right Coronary Artery Related Acute Inferior Wall Myocardial Infarction in Patients Undergoing Adjunctive Angioplasty after Fibrinolysis

**DOI:** 10.5681/jcvtr.2014.022

**Published:** 2014-06-30

**Authors:** Bahram Sohrabi, Ahmad Separham, Reza Madadi, Mehrnoush Toufan, Nasibeh Mohammadi, Naser Aslanabadi, Babak Kazemi

**Affiliations:** Cardiovascular Research Center, Tabriz University of Medical Sciences, Tabriz, Iran

**Keywords:** Inferior Wall Myocardial Infarction, Fibrinolysis, Balloon Angioplasty, Treatment Outcome

## Abstract

***Introduction:*** Prognostic differences between anterior and inferior wall Myocardial Infarction (MI) has been extensively investigated, but there is limited information about similar comparison between inferior wall MI caused by right coronary artery (RCA) and left circumflex artery (LCX) occlusion. The aim of present study was to compare prognostic differences between LCX- and RCA-related acute inferior wall ST-segment elevation MI (STEMI) treated by routine adjunctive angioplasty after receiving thrombolytic therapy (TLT).

***Methods:*** Between March 2012 and June 2013 one hundred fifty consecutive patients with acute inferior wall STEMI were studied. Patients were divided into two groups according to the infarct related artery (LCX vs. RCA). All patients underwent routine adjunctive angioplasty after TLT during the index hospitalization and clinical characteristics and outcomes were compared.

***Results:*** RCA and LCX arteries were occluded in 97 (64.7%) and 53 (35.3%) of patients, respectively. Two groups were similar in baseline characteristics except multiple-vessel disease was more prevalent with LCX occlusion (p= 0.008). There was a higher cardiac enzyme release (p< 0.001), more significant mitral regurgitation (MR) (p= 0.015), and lower left ventricular ejection fraction (LVEF) (p= 0.01) in patients with LCX occlusion. Multivariate analysis showed cTn-I release, occurrence of MR, and lower LVEF as independent factors leading to poor outcome.

***Conclusions:*** There were higher cTn-I release, MR occurrence, and lower LVEF in LCX-related acute inferior wall STEMI, all associated with poor outcome. Therefore, patients with ECG finding in favour of LCX occlusion should be considered as high risk and an invasive approach should be planned.

## Introduction

Acute myocardial infarction (MI) is caused by plaque rupture in one of the major epicardial coronary arteries.^1^ The prognostic outcome between anterior and inferior wall MI has been extensively investigated. Nienhuis et al.^2^ showed more favorable short and long-term clinical outcomes for inferior compared to anterior MI. The extent of myocardial damage in acute left anterior descending artery (LAD) occlusion is commonly larger than in either acute right coronary (RCA) or left circumflex (LCX) artery occlusion simply because it perfuses a larger myocardial territory. Limited information exists about similar comparison between inferior wall MI caused by RCA and LCX occlusion. On the other hand, the prognosis of LCX- related MI is less clear. In large randomised trials on ST-segment elevation myocardial infarction (STEMI) less than 20% of cases had LCX as the culprit, many missed due to limited or absent ST-segment elevation.^3,4^ Rasoul et al.^5^ showed that enzymatic infarct size was significantly higher and Left ventricular ejection fraction (LVEF) significantly lower in patients with LCX- related MI treated by primary percutaneous intervention (PCI). However, most patients with acute STEMI cannot receive timely primary PCI because of lack of facilities or delays in patient transfer or catheterization team mobilization. The advantages of TLT include easy administration, widespread availability and early patency of the infarct-related artery (IRA). However, complete reperfusion is only achieved in 60% of patients, who remain at risk for reocclusion, reinfarction, and recurrent ischemia.^6^ Even if it is likely that TLT will be successful (ST-segment resolution >50% at 60–90 min; typical reperfusion arrhythmia; disappearance of chest pain), a strategy of routine early angiography is recommended if there are no contraindications.^7^ A crucial issue is the optimal delay between lysis and PCI and based on the available data, a time window of 3–24 h after successful lysis is recommended.^7^ This time window is justified by the concern of thrombotic complications when an intervention is performed immediately following the administration of the lytic agent due to its prothrombotic effects and, on the other hand, of spontaneous re-infarction in the first day following thrombolysis.^8^

Based on this information, we conducted this study to compare the prognostic outcomes between LCX- and RCA-related acute inferior wall STEMI, using the strategy of routine PCI after TLT during the index hospitalization.

## Materials and methods

One hundred and fifty eligible consecutive patients between March 2012 and June 2013 were enrolled into this cohort study. Permission to conduct the study was obtained from the Ethics Committee at Tabriz University of Medical Sciences. All patients signed an informed consent to participate in study. Acute inferior STEMI was defined as: typical chest pain lasting for >30 minutes with ST-segment elevation >1 mm in 2 consecutive inferior leads. All patients received standard dose reteplase (10 IU intravenous bolus over 2 min and repeated at 30 minutes) and routinely underwent selective coronary angiography by the trans-femoral approach before hospital discharge. We used Judkins guiding catheter (Cordis, Warren, NJ, USA) for the RCA and the XB guiding catheter (Cordis, Warren, NJ, USA) for the LCX intubation. Following coronary angiography, all patients with residual stenosis of at least 70% in the IRA underwent PCI, regardless of flow and patency status. Treatment of IRA with stenosis of 50–70% and thrombus, ulceration or spontaneous dissection was left to the judgment of the interventional cardiologist. Stents were used whenever technically possible. The use of drug-eluting stents was encouraged, although other approved bare metal stents were permitted. Referral for surgery was recommended in the presence of significant left main coronary artery disease, serious 3-vessel disease, or severe MR. Baseline characteristics, type of stent, and multiple-vessel disease presences were recorded in two groups. Multiple vessel disease was defined as stenosis ≥50% in ≥2 major epicardial coronary arteries. Prognostic outcomes including peak level of CPK-MB, cTn-I, LVEF, heart failure (HF) during hospitalization, severity of MR, atrioventricular block, re-hospitalization, and 30-day mortality in two groups of patients were collected prospectively. HF was defined as New York Heart Association functional class ≥3. All patient received Clopidogrel (300 mg), aspirin (325 mg), atrovastatin (40 mg). Beta-blockers, angiotensin converting enzyme inhibitors, and diuretics were used as clinically required.

### Statistics

Data were expressed as mean ± SD (Standard Deviation) or as number (percent). Comparisons in continuous variables were done by independent t test and categorical variables by Chi Square test (or Fisher Exact Test if applicable). Multiple stepwise logistic regressions were used for comparison of prognostic outcomes in two groups of patients. Statistical analysis was performed using SPSS for Windows version 16 (SPSS Inc., Chicago, IL). A 2- sided p-value <0.05 was considered statistically significant.

## Results

The mean age of the patients was 55.8 ± 10.5 years, and 89.3% were male. RCA occlusion occurred in 97 (64.7%) patients, whereas the remaining 53 (35.3%) had LCX occlusion. The mean time from onset of chest pain to TLT (3 hours) and the mean time to PCI (2 days) were similar between groups. Table 1  shows the comparison of baseline characteristics of the study groups. As shown in this table two groups of patient were similar in all baseline characteristics as age, gender, hypertension, current smoking, hyperlipidemia, systolic and diastolic blood pressure, creatinine level, white blood cell count, blood sugar, presence of diabetes mellitus and type of stent, but multiple-vessel disease was significantly more prevalent in the LCX group (p= 0.008). So, in comparison of prognostic outcomes of patients with RCA- and LCX-related acute inferior wall MI, multiple-vessel disease should be considered as a strong confounder. Comparison of prognostic outcomes of patients with RCA- and LCX-related acute inferior wall MI is shown in table 2 . As shown, the LCX group had significantly more myocardial enzyme release (p< 0.001), lower LVEF (p= 0.01), more HF (p=0.001), and more significant MR (p= 0.015) than the RCA group. Complete heart block, re-hospitalization and mortality at 30-days were similar in both groups. A Multiple Stepwise Logistic Regression model was used to evaluate the prognostic outcomes of LCX- vs. RCA-related acute inferior wall MI such as peak level of CPK-MB, cTn-I, LVEF, HF, significant MR and multiple-vessel disease. The results of this analysis indicate that cTn-I (OR= 1.04), LVEF (OR= 0.76) and significant MR (OR= 4.88) were prognostic outcomes associated with LCX-related acute inferior wall MI (Table 3). After adjusting for other factors, patients with LCX- vs. RCA-related acute inferior wall MI had higher cardiac cTn-I, significant MR and LVEF (Table 3).

**Table 1:** Comparison of baseline characteristics for patients with RCA- and LCX-related acute inferior wall MI.

**Variables**	**LCX group** **(n=53)**	**RCA group** **(n=97)**	**P-value**
Age (years)	57.6 ± 10.9	54.8 ± 10.1	0.130
Gender (% male)	47 (88.7%)	87 (89.7%)	0.848
Hypertension (%)	22 (41.5%)	40 (4%)	0.902
Current smoking (%)	26 (50.0%)	59 (62.8%)	0.134
Hyperlipidemia (%)	17 (32.7%)	28 (30.1%)	0.747
Systolic blood pressure (mm Hg)	129.2 ± 23.9	122.8 ± 20.7	0.096
Diastolic blood pressure (mm Hg)	79.8 ± 19.4	77.6 ± 15.5	0.453
Creatinine level (mg/dL)	1.13 ± 1.41	0.94 ± 0.20	0.329
White blood cell count (×1000/ mL)	11.7 ± 3.4	11.7 ± 3.2	0.954
Blood sugar (mg/dl)	164 ± 80.2	162 ± 82.7	0.910
Diabetes mellitus (%)	12 (22.6%)	22 (23.4%)	0.916
Stent BMS/DES (%)	35 (70.0%)	63 (70.0%)	1.000
Multiple-vessel disease (%)	34 (65.4%)	41 (42.7%)	0.008

**Table 2:** Comparison of prognostic outcomes of the patients with RCA- and LCX-related acute inferior wall MI.

**Variables **	**LCX group** **(n=53)**	**RCA group** **(n=97)**	**P-value**
Peak level of CPK-MB (IU/L)	246.7 ± 132.3	161.0 ± 105.0	<0.001
Cardiac troponin I (ng/ml)	27.5 ± 11.2	14.7 ± 13.2	<0.001
Complete Heart Block (%)	1 (2.0%)	5 (6.0%)	0.411
Left ventricular ejection fraction (%)	43.3 ± 6.5	55.8 ± 45.9	0.010
Advanced heart failure (%)	10 (20.0)	2 (2.4%)	0.001
Significant mitral regurgitation (%)	18 (34.0%)	16 (16.5)	0.015
Re-hospitalization (%)	4 (7.5%)	5 (5.2%)	0.721
30-day mortality (%)	3 (5.7%)	4 (4.1%)	0.698

**Table 3:** Multiple Stepwise Logistic Regression model of prognostic outcomes of LCX- vs. RCA-related acute inferior wall MI.

**Variables **	**Odds ratio**	**Confidence Interval 95%**	**P-value**
Peak level of CPK-MB (IU/L)	1.0	0.99 – 1.08	0.400
Cardiac troponin I (ng/ml)	1.04	1.01 – 1.08	0.021
Left ventricular ejection fraction (%)	0.76	0.68 – 0.86	<0.001
Advanced heart failure (%)	2.44	0.16 – 16.2	0.517
Significant mitral regurgitation (%)	4.88	1.62 – 13.43	0.003

## Discussion

The aim of the present study was to compare prognostic outcomes between LCX- and RCA-related acute inferior wall MI. Baseline characteristics were comparable between groups. Univariate analysis showed that the LCX group had significantly higher cardiac enzyme release, lower mean LVEF, and more significant MR and HF events than the RCA group. Furthermore, although complete heart block, re-hospitalization and 30-day mortality occurred more frequently in the LCX group, the difference between groups did not reach statistical significance.

Our results were similar to previous studies. Yip et al.^8^ have previously shown that patients with inferior wall MI caused by dominant LCX occlusion had an unfavorable clinical outcome. Chen et al.^9^ have shown that the 30-day prognostic outcome was less favorable in LCX- compared to RCA-related inferior STEMI undergoing primary PCI. Rasoul et al.^5^ showed that infarct size was significantly higher and LVEF< 45% was more often present in the LCX- compared to RCA-related inferior MI.

All these previous studies were done with the main reperfusion strategy being primary angioplasty. In this study we compared the prognostic outcomes between patients with LCX- and RCA-related acute MI treated with TLT and early adjunctive angioplasty before discharge. This study emphasizes the fact that LCX-related infarction has also a worse prognosis compared with RCA-related infarction when treated with TLT and early adjunctive angioplasty.

It is, however, important to know the limitations of the study. The sample size of present study was not sufficient to compare complete heart block, re-hospitalization and 30-day mortality in two groups.

## Conclusion

The prognostic outcomes were less favorable in patients with LCX-related compared with RCA-related inferior STEMI. Awareness of this distinctive clinical feature is crucial in our daily clinical practice and could finally improve the outcomes of this subset of patients. Therefore, patients with inferior MI that ECG finding suggests LCX involvement should be considered as high risk and an invasive approach should be pursued.

## Acknowledgments

This article was written based on a dataset of fellowship in interventional cardiology thesis, registered in Tabriz University of Medical Sciences.

## Ethical issues

Permission to conduct the study was obtained from the Ethics Committee at Tabriz University of Medical Sciences.

## Competing interests

Authors declare no conflict of interest in this study.
